# Closing the yellow gap with Eu- and Tb-doped GaN: one luminescent host resulting in three colours

**DOI:** 10.1038/s41598-022-06148-0

**Published:** 2022-02-15

**Authors:** Cordula Braun, Liuda Mereacre, Zheng Chen, Adam Slabon

**Affiliations:** 1 Institute for Applied Materials (IAM), Karlsruhe Institute of Technology (KIT), Herrmann-von-Helmholtz-Platz 1, 76344 Eggenstein-Leopoldshafen, Germany; 2 Institute of Inorganic Chemistry, RWTH Aachen University, Landoltweg 1, 52056 Aachen, Germany; 3 Department of Materials and Environmental Chemistry, Stockholm University, Svante Arrhenius väg 16 C, 106 91 Stockholm, Sweden

**Keywords:** Chemistry, Materials chemistry, Materials for optics, Lasers, LEDs and light sources

## Abstract

Gallium nitride (GaN) is a key material when it comes to light-emitting diodes (LEDs) and has pushed the LED revolution in lighting and displays. The concept of down-conversion of a GaN-based blue LED offers the possibility to provide efficient generation of monochromatic, high-color purity light resulting in a highly efficient warm-white all-nitride phosphor-converted light emitting diode (pc-LED). Although the down conversion of blue light from InGaN LEDs has become a dominant technique for producing white light, there are still some technical challenges, e.g. the immiscibility of GaN and InN and the lattice mismatch between the substrate and InGaN, that have to be overcome. Here we demonstrate the doping of bulk GaN with europium, terbium and the combination of both resulting in intriguing luminescence properties, pushing the role of GaN:Eu,Tb as a chief component in future light emitting diodes. This colour tuning proves that one luminescence host can provide three colours (red, green and orange) and that even the so called “yellow gap” could be closed with a III-nitride. By using one material for all colours, it will be possible to overcome the technical challenges in building up LED devices, which will open up new capabilities for modern highly efficient phosphors.

## Introduction

Gallium nitride (GaN) has set the benchmark in the last decades when it comes to light-emitting diodes (LEDs). As a key material, it has driven the LED revolution in lighting and displays. The lighting industry with its various market segments, including automotive lighting, indoor and outdoor lighting, medical applications, lifestyle products and vertical farming has arisen from these fundamental discoveries enabled by the exploration of light's interaction with matter^1,2^. The first light emission from a solid-state material driven by an electric current was reported in 1907 by *H.J. Round*^3^ and this outreaching discovery paved the way of electroluminescence and highly efficient phosphors in modern LED technology. This investigation triggered further studies on the optoelectronic processes taking place in semiconductors based on the recombination of electric charges^4,5^. The first GaAsP LEDs was reported by Craford et al*.*^6,7^ in 1971 and 1972, but many technical challenges still were to overcome. The development of metal-organic vapour phase epitaxy (MOVPE) technique led the foundation for all future milestones to come. The final breakthrough came from the investigations of Nakamura and coworkers^8–10^, making p-doping of GaN and its ternary alloys (InGaN, AlGaN) easily accessible by a post-growth thermal annealing treatment and launched the invention of highly efficient blue-emitting diodes.

In 2014, the Nobel Prize in Physics was awarded to Isamu Akasaki, Hiroshi Amano and Shuji Nakamura for “the invention of efficient blue light-emitting diodes which has enabled bright and energy-saving white light sources”^11^. White light from LEDs can be build up combining a red, green and blue emitting semiconductor or by phosphors down-converting the emission of short wavelength emitting GaN/InGaN LEDs^12–14^.

One of the challenges here is the comparatively low external quantum efficiency (EQE) of the green-emitting semiconductors, because of the lattice mismatch between the substrate and InGaN, causing high defect density, immiscibility of GaN and InN^15^ or chemical interaction with packaging materials. As such, there is an urgent necessity of developing a solid-state material with a very narrow emission bandwidth in the green spectral region^14^. The concept of down conversion of a GaN-based blue LED offers also the possibility to provide efficient generation of monochromatic, high-color purity light resulting in a highly efficient warm-white all-nitride phosphor-converted light emitting diode (pc-LED). Eu^2+^-doped nitridosilicates and oxonitridosilicates emerged as important host lattices for phosphor-converted light-emitting diodes (pc-LEDs) due to their very high chemical and thermal stability, their very high quantum efficiency of the luminescence process and their very low thermal quenching^13,16–26^. Namely M_2_Si_5_N_8_:Eu^2+^ and MSi_2_O_2_N_2_:Eu^2+^ (M = alkaline earth) have been employed as highly effective red–orange (2-5-8) and yellow–green (1-2-2-2) phosphors, respectively. Even the so called “yellow gap”, neither III-nitrides nor III-phosphides had been able to close, could be bridged with (Ba,Sr)_2_Si_5_N_8_:Eu^2+^^14^. Just recently, new Mg-nitridosilicates emerged as next generation red phosphor materials with superior luminescence properties and exceptionally narrow red emission^27–29^.

Many investigations concerning MOVPE-grown GaN, GaN nanowires/ nanocrystals, epilayers/thin layers, GaN quantum dots doped with Eu or in some cases with Tb, can be found in literature^27–40^. (and references herein) But definitely Eu was not combined together with Tb. And as we know that there exist so many epitaxial layers and thin film studies we consciously wanted to differentiate and have used bulk material of GaN for our studies. However investigations about Eu doped GaN and several co-dopants have been made. Photoluminescence properties of Er, Eu, Tm-doped GaN thin-films prepared by solid-source molecular beam epitaxy were studied by Hömmerich et al*.*^44^. Three-color integration on Tm, Er, and Eu doped GaN electroluminescent thin films was realized by Wang and Steckl^45^. But here it is important to notice as well that the dopants do not have been combined and it refers about electroluminescence and not photoluminescence. Mitchell et al*.*^46^ have demonstrated that the emission from a GaN:Eu LED can be tuned from red to yellow under current injection. They describe as well the co-doping of GaN with Si and Mg where new Si-Mg related Eu complexes were observed with a significantly enhanced energy transfer efficiency. But here again it´s not about photoluminescence. Mg doping in relation with GaN:Eu has been investigated by several other groups as well^47–51^, as the Mg co-doping is supposed to increase the photoluminescence (PL) as well as the electroluminescence intensity. Hoang^52^ investigated defect physics of Eu-doped GaN using first-principles hybrid density-functional. His calculations are taking into account as well the interaction between Eu and other ions as O, Si, C, H, and Mg. But here as well Eu and Tb are not incorporated into the host material.

To cover the colour range from blue, over green and orange to red (ca. 450 to 650 nm), a combination of InGaN und AlGa(In)P is necessary. Several challenges have to be addressed here. The decrease of the external quantum efficiency versus emission wavelength around 560 nm, is termed the “yellow gap”^14^. The immiscibility of GaN and InN leads to a reduction in performance of InGaN-based LEDs with higher InN mole fractions^14^. The lattice mismatch between GaN, InN, GaAlN, AlGaP, different thermal expansion coefficients and the variations of the In/Al content decrease the luminescence performance tremendously. Down conversion of blue light from InGaN LEDs by suitable color converters, especially phosphors, has become a dominant technique for producing white light, but still some technical challenges have to be overcome. One point here are structural misfits of thin films because of the different host materials. Using one bulk material (GaN) for all colours (blue, red, green, orange) could enable therefore the next step of modern high performance GaN LEDs.

Here, we demonstrate the doping of bulk GaN with europium and terbium and the combination of both results in intriguing luminescence properties of all three doped compounds. This renders GaN:Eu,Tb^53^ as a prospective chief component in future light emitting diodes (LEDs).

## Results and discussion

### Synthesis

**Figure 1:**
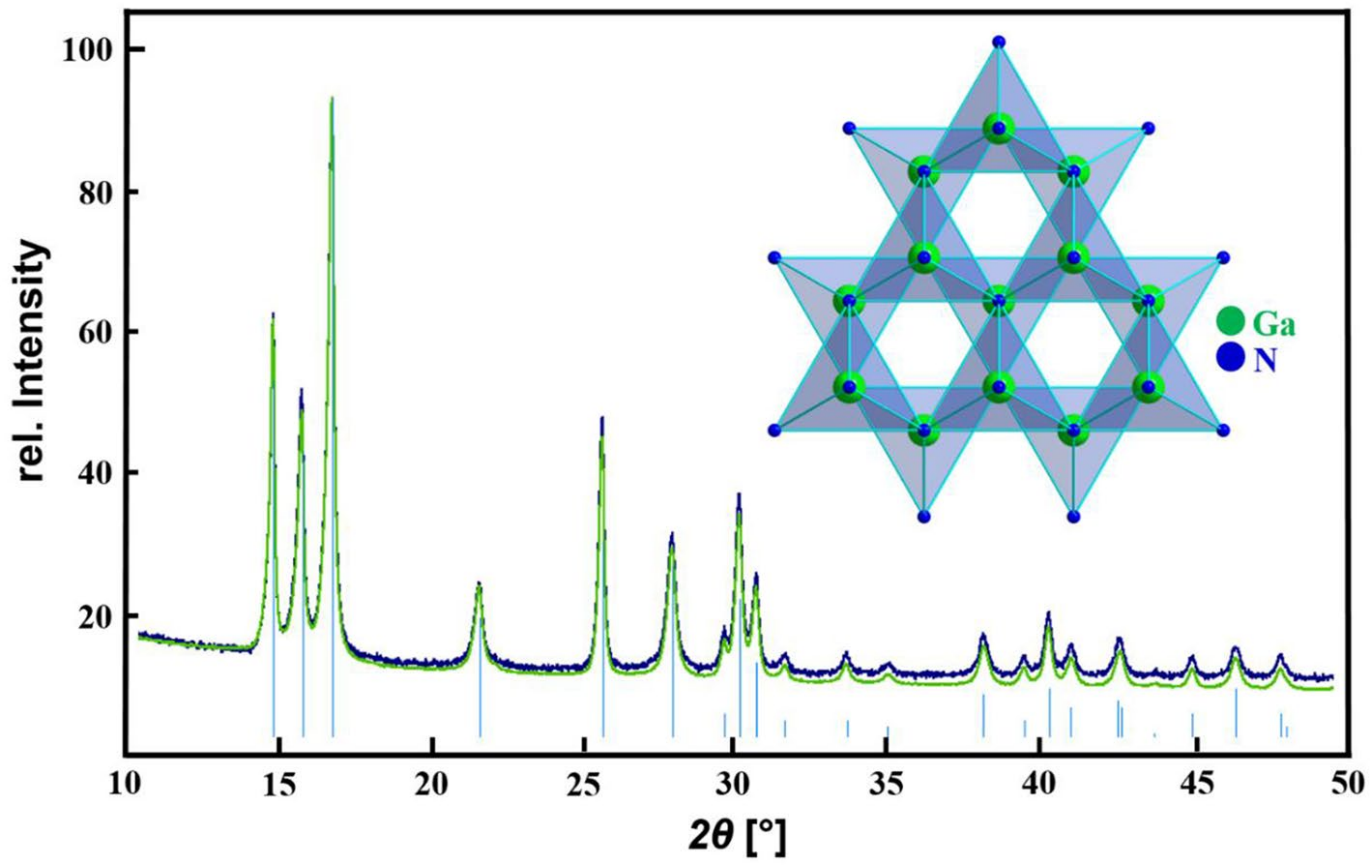
X-ray powder diffraction patterns of undoped GaN (blue), GaN:Tb (green) and GaN ICSD [50-07920] (bright blue), (*λ* = 0.709026 Å), the inset shows the characteristic motif of the underlying GaN structure.

By using various synthetic approaches (e.g. low temperature urea-based method) we succeeded to co-dope GaN (Chempur 99,999%) with MCl_3_ × 6 H_2_O or M(NO_3_)_3_ × 5H_2_O (M = Eu, Tb) as substitutes (see Fig. 1). For a comparison of the powder diffraction pattern of GaN:Eu^3+^ and GaN:Tb^3+^ see supplement Figure S1. SEM EDX measurements of GaN confirmed the atomic ratio of Ga:N of 1:2 and an europium and terbium content of 3–5% was found, but no oxygen within the detection limit.

### Luminescence properties

Eu^3+^ is considered as one of the most important activator ions with red emission corresponding to the transition ^5^D_0_–^7^F_J_ (*J* = 1–6). The green emission of Tb^3+^ is due to the transition between the emitting states of ^5^D_J_ and the excited states of ^7^F_J_. The main intense green emission is attributed to the transition of ^5^D_4_–^7^F_5_, which is located at approx. 544 nm. A relevant point here is that normally the doping ion is inserted during the main synthesis and not afterwards. In general, doping is performed with Eu^2+^ and only one activator ion is used. Consequently, one host can only provide one colour. Since different aspects come into play when we think about mixing colours within one host, such an approach for colour tuning offers tremendous opportunities for highly efficient phosphors.

**Figure 2:**
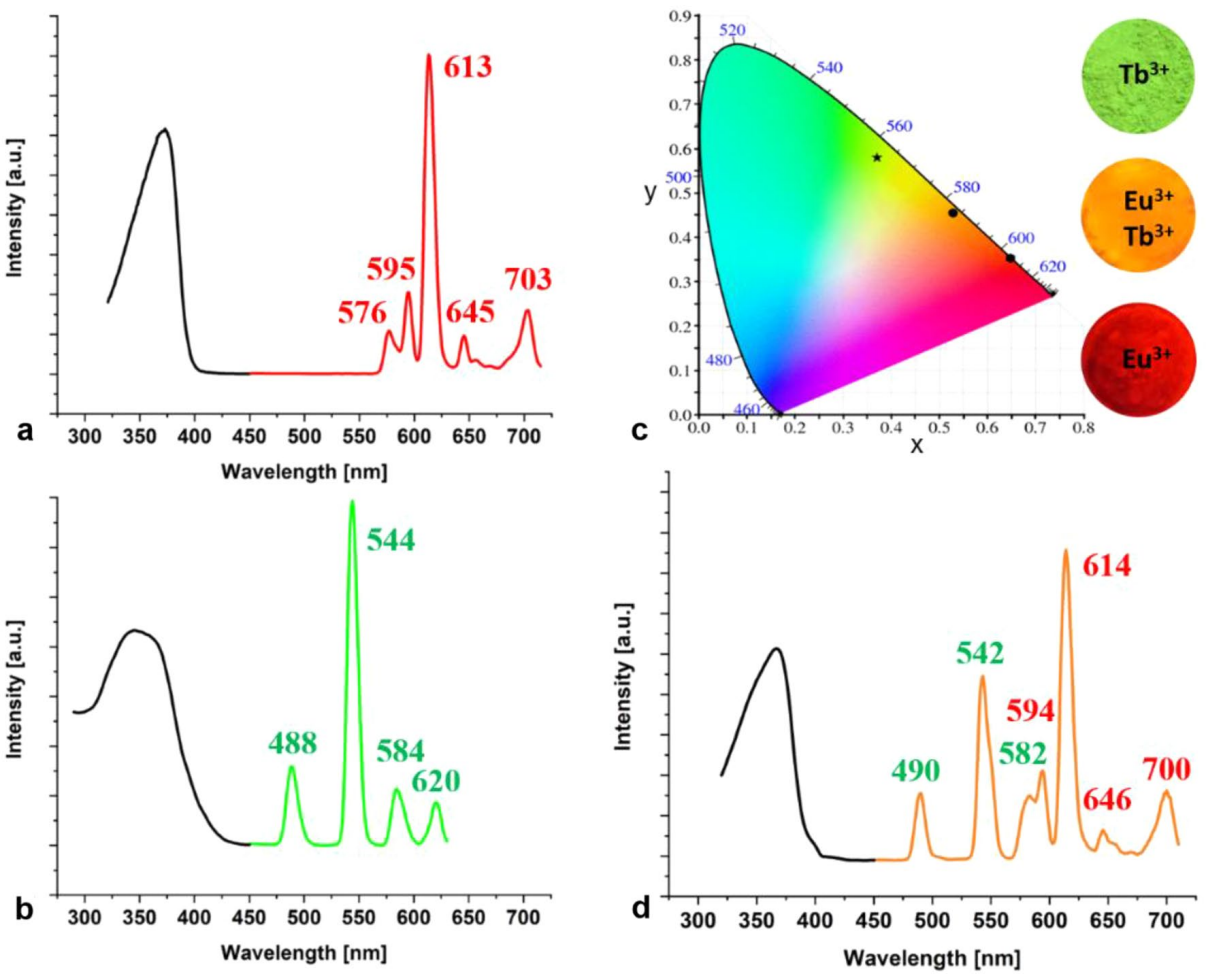
Excitation (black) and emission spectra (coloured) of GaN doped with (**a**) Eu^3+^ (red, filled hexagonal), (**b**) Tb^3+^ (green, filled star) and (**d**) Eu^3+^ and Tb^3+^ (orange, filled circle), (**c**) CIE 1931 diagram of GaN doped with Tb^3+^, Eu^3+^,Tb^3+^ and Eu^3+^.

In this work, we doped GaN with Eu^3+^ and Tb^3+^ as activator ions, with each individually and with both simultaneously. Figure 2c shows the CIE 1931 diagram and the colours of doped GaN, proving that a colour tuning of one host with different activator ions and their combination is possible. Having a closer look at the CIE and the basics of colour mixing, it is clear that the combination of green and red leads to orange. (see Fig. 2d) This effect could be proven in the meantime for other doped nitrides and carbodiimides as well^53,54^.

GaN: Eu^3+^ shows strong characteristic emission peaks of Eu^3+^ within the region of 570–700 nm, indicating an energy transfer between the GaN host and the Eu^3+^ ions. The peaks associated with the intra-4f shell transitions (^5^D_0_–^7^F_J_) of the Eu^3+^ ions, are the peaks at 576, 595, 613 and 645 nm, wherein the one at 613 nm is the strongest (see Fig. 2a). Peng et al*.*^37^assumed according to extended x-ray absorption fine structure measurements, that the Eu^3+^ ion substitutes the Ga site, which is in accordance with our assumption.

The excitation (monitored at 544 nm) and emission spectra of Tb^3+^ doped GaN are represented in Fig. 2b. The excitation spectrum exhibits a broad and intense band in the range from 290 to 380 nm with a peak at around 322 nm. This broad band is attributed to 4f^8^–4f^7^5d^1^ transition of the Tb^3+^ ions. The strongest emission peak is at 544 nm with a **F**ull **W**idth at **H**alf **M**aximum (FWHM) ~ 12 nm corresponding to the ^5^D_4_ → ^7^F_5_ transition, while the peaks at 488 nm, 584 nm and 620 nm, respectively, originate from the ^5^D_4_ → ^7^F_6_, ^5^D_4_ → ^7^F_4_ and ^5^D_4_ → ^7^F_3_ transitions of the Tb^3+^ ions. Until now, green emitting phospors could only be realized as oxynitrides (e.g. β-SiAlON:Eu^2+^^55^) or oxonitridosilicates (e.g. SrSi_2_O_2_N_2_:Eu^2+^^56,57^). The here presented luminescence behavior of GaN:Tb^3+^ shows that green emitting phosphors^53^ can be also achieved with purely nitridic compounds.

The energy level distributions of Tb^3+^ and Eu^3+^ have a large overlap and their energy transfer has been proven to be very effective^58^. The blue-green light of the Tb^3+^ transition (^5^D_4_ → ^7^F_6_,_5_) is emitted by polychromatic relaxation and the energy is transferred to the ^5^D_1_ and ^5^D_0_ levels of the Eu^3+^ by cross relaxation. The Eu^3+^ ions absorb the energy from Tb^3+^ and emit therefore orange light.

Figure 2d proves that it is possible to insert two different activator ions in one host showing the typical bands of Eu^3+^ as well as the ones of Tb^3+^ in one spectrum resulting in a saturated orange body colour. However a close look reveals that this is not only a superposition of the Eu^3+^- and the Tb^3+^-spectrum of GaN, because this orange spectrum differs clearly concerning peak form, intensity and wavelength. Here it becomes evident, that this orange colour is only possible by mixing the ions on an atomic scale and cannot be realized by a mixture of particles of the red and the green doped GaN. To prove this GaN:Eu and GaN:Tb (same molar ratio Eu:Tb as in GaN:Eu,Tb) have been mixed in a mortar. It becomes obvious that this does not result in an orange luminescent GaN. Instead the red GaN:Eu is dominant in the mixture, resulting in the same red colour coordinates of just GaN:Eu. See Figure S10. The FWHM point out the same values as in the single dopings. The line widths of the emission spectra (FWHM) of very narrow banded nitridosilicate phopshors range between 35 and 50 nm^2^, of nitride-based LEDs they vary typically between 20 and 35 nm and of phosphide-based ones between 15 and 25 nm^12^. Here we range from 8 to 12 nm for GaN:Eu^3+^,Tb^3+^ which fits perfectly to the respecting GaN spectra in literature. The CIE values of amber emitting Phosphor GaN:Eu^3+^/Tb^3+^ x,y = 0.528, 0.454 (see Fig. 2c) are very similar to those of (Ba,Sr)_2_Si_5_N_8_:Eu^2+^^14^(x,y = 0.579, 0.416) and are lying therefore well within the “amber box” of the SAE specifications^14^. This 2-5-8 nitridosilicate phosphor has been considered as an important breakthrough for bridging the “yellow gap”^14^, and is established nowadays in numerous optoelectronic applications.

To elucidate if the Eu,Tb ions really have been inserted into the GaN structure and the luminescence does not result from the respective doping compounds a comparison of the luminescence spectra of GaN:Eu^3+^ and EuCl_3_ × 6 H_2_O is shown in Figure S1. As optical and luminescence spectra are highly sensitive to structural deformation of the nearest environment of rare-earth (*RE*) ions, it is clearly evident that the spectrum has changed due to the insertion of the Eu cation into the structure of GaN. Furthermore, very detailed comparisons of several Eu- and Tb-doped GaN thin films and possible side products have been carried out (see supplement Figures S2-9). Here it becomes obvious that some luminescence spectra seem very similar, but when taking a closer look, they clearly differ. According to De Boer et al.^31^, sample growth and doping conditions play as well an important role in influencing the PL (photoluminescence) spectra.

### Mott–Schottky (MS) measurements

**Figure 3:**
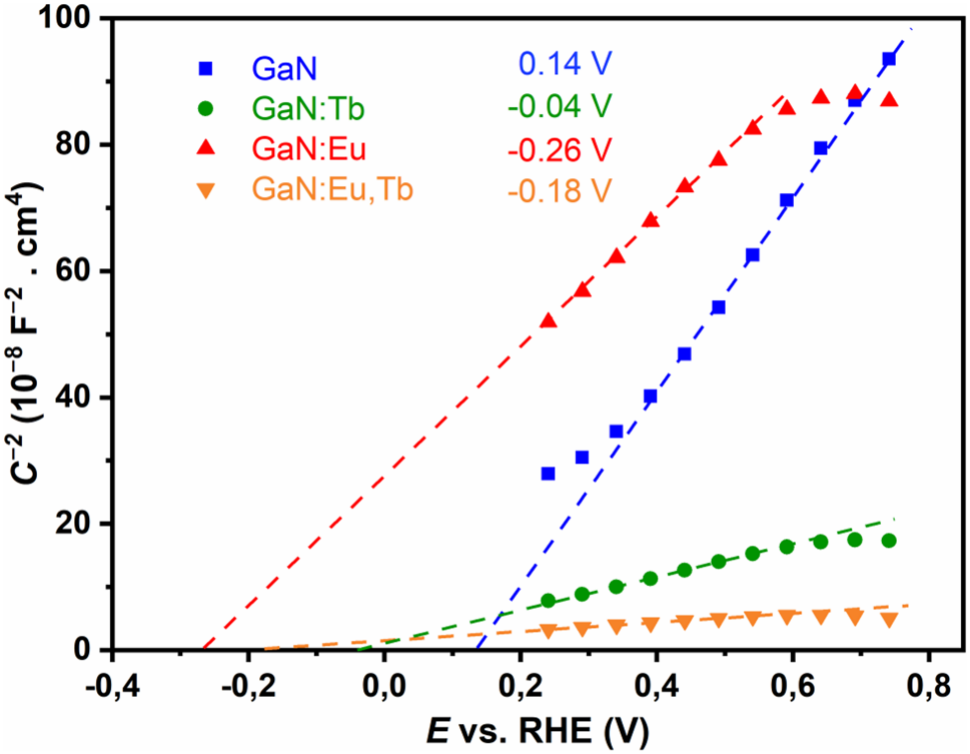
Mott-Schottky (MS) analysis of Electrochemical Impedance Spectroscopy (EIS) measurements of (**a**) undoped GaN (blue), GaN:Tb^3+^ (green), GaN:Eu^3+^ (red) and GaN:Eu^3+^,Tb^3+^ (orange); (Measurements were performed in a 0.1 M potassium phosphate electrolyte at an applied frequency of 10 Hz). The extrapolated curves at y = 0 correspond to the conduction band edges, because the materials exhibit a positive slope that is characteristic for an n-type semiconductor. The determined flatband potentials are provided in the inset.

Electrochemical Impedance Spectroscopy (EIS) is an appropriate tool to study ion diffusion and to resolve the chemical identity of the charge carriers by the use of blocking electrodes. Mott–Schottky (MS) measurements are a very sensitive technique to probe changes in the electronic band structure, i.e. charge carrier density, type of semiconducting behaviour and band edge positions, upon doping. For the elucidation of doping effect on the electronic properties of GaN, Mott-Schottky (MS) measurements were conducted for the bare and *RE*-doped samples. They were performed in a 0.1 M potassium phosphate electrolyte at an applied frequency of 10 Hz. As shown in Fig. 3, all of the acquired curves show a positive slope corresponding to the characteristic of n-type semiconductors^59^. In addition, a smaller slope for all curves can be attributed to increased charge carrier density for GaN upon doping with the rare earth metals^60,61^. The extrapolation of the curves allows to estimate the flat band potential as the corresponding conduction band edge of n-type semiconductor. The obtained conduction band edges reveal that *RE*-doped GaN yield a significant negative shift, indicating *RE* doping can be applied to modify the (photo)electrochemical properties of GaN^61–63^.

## Conclusion

We demonstrated the doping of bulk GaN with europium and terbium and the combination of both resulting in intriguing luminescence properties of all three doped compounds (red, green, orange), rendering GaN:Eu,Tb as an prospective component in future light emitting diodes (LEDs). Our results highlight the opportunities for controlling functionality and luminescence properties of modern energy-efficient white light-emitting diodes and energy-efficient power electronic devices. Especially the closing of the “yellow gap” is a big step foreward.

The here presented methodology of doping bulk GaN applied may be generalized to create different dimensions of device architectures for LEDs, as the possibility to modify their charge transport properties by introducing dopant atoms has turned out for some time to be crucial for the performance of inorganic LEDs.

## Experimental section

### Doping of GaN

The doping of GaN (Chempur 99,999%) was realised by a successive combustion synthesis.

Briefly, the respective metal chlorides (MCl_3_·6 H_2_O or M(NO_3_)_3_·5H_2_O (M = Eu, Tb), NH_4_NO_3_, Urea and H_2_O were added to the sample. This mixture was put into an oven at 400–600° for 10 min. The doping, which is about 3–5%, has been checked via EDX measurements.

### X-ray diffraction

#### Powder Diffraction

X-ray diffraction experiments on powder samples of GaN were performed on a STOE STADI P powder diffractometer in Debye–Scherrer geometry with Ge(111)-monochromatized Mo-*Kα*_1_ radiation (*λ* = 0.709026 Å). The sample was enclosed in a glass capillary of 0.3 mm diameter.

### EDX measurements

SEM was performed on a Zeiss Merlin microscope and for EDX we used a Quantax 400 system from Bruker.

### Mott–Schottky (MS) measurements

#### Electrodes fabrication

GaN and the corresponding rare earth metal (RE, RE = Eu^3+^, Tb^3+^ and Eu^3+^/Tb^3+^) doped semiconductor electrodes were prepared by electrophoretic deposition. Fluorine doped tin oxide (FTO) glass (2.2 mm thick, Sigma-Aldrich) was used as the substrate after sequentially ultrasonic cleaning with dilute nitric acid, acetone and ethanol for 15 min. The dispersion was prepared by mixing 5 mg iodine and 20 mg sample with 20 ml acetone, followed by treatment with ultrasounds. The electrodes were obtained after depositing the dispersed powder at 30 V and drying under ambient atmosphere.

#### Mott–Schottky (MS) measurements

The MS measurements were performed in an electrochemical cell using a potentiostat (Gamry instruments) operating in a three-electrode setup. The deposited samples on FTO, a 1 M Ag/AgCl electrode and a platinum wire were used as a working electrode, a reference electrode and a counter electrode, respectively. All MS data were recorded *vs. E*_1 M Ag/AgCl_ (V), which was subsequently converted with respect to *E*_RHE_ (V) according to the formula: *E*_RHE_ (V) = 0.235 + *E*_1 M Ag/AgCl_ + [0.059 × pH] (V) at 25 °C.

#### Luminescence

The luminescence spectra and quantum yield measurements were performed on a Fluorolog®-3 Horiba Jobin Yvon equipped with a TBX detector picosecond photon detection device and a 450 W xenon lamp.

## Supplementary Information

Supplementary Information.
