# Supplementary material for: Closing the yellow gap with Eu- and Tb-doped GaN: one luminescent host resulting in three colours
Source: Sci Rep. 2022 Feb 15;12:2503. doi: 10.1038/s41598-022-06148-0 (PMC8847369; doi:10.1038/s41598-022-06148-0)
Supplement: Supplementary file 1 — Supplementary Information. [file 41598_2022_6148_MOESM1_ESM.docx]

**Supplement**

**Closing the yellow gap with Eu- and Tb-doped GaN: One luminescent host resulting in three colours**

Cordula Braun,^[a]*^ Liuda Mereacre,^[a]^

Zheng Chen,^[b]^ and Adam Slabon^[c]^

[a] ^*^ Dr. C. Braun, L. Mereacre,

Karlsruhe Institute of Technology (KIT)

Institute for Applied Materials (IAM)

Herrmann-von-Helmholtz-Platz 1,

D-76344 Eggenstein-Leopoldshafen

E-mail: [Cordula.Braun@kit.edu](mailto:Cordula.Braun@kit.edu)

[b] Z. Chen,

Institute of Inorganic Chemistry, RWTH Aachen University,

Landoltweg 1,

D-52056 Aachen

[c] Prof. Dr. A. Slabon,

Department of Materials and Environmental Chemistry,

Stockholm University,

Svante Arrhenius väg 16 C,

106 91 Stockholm, Sweden

*Figure(s)*


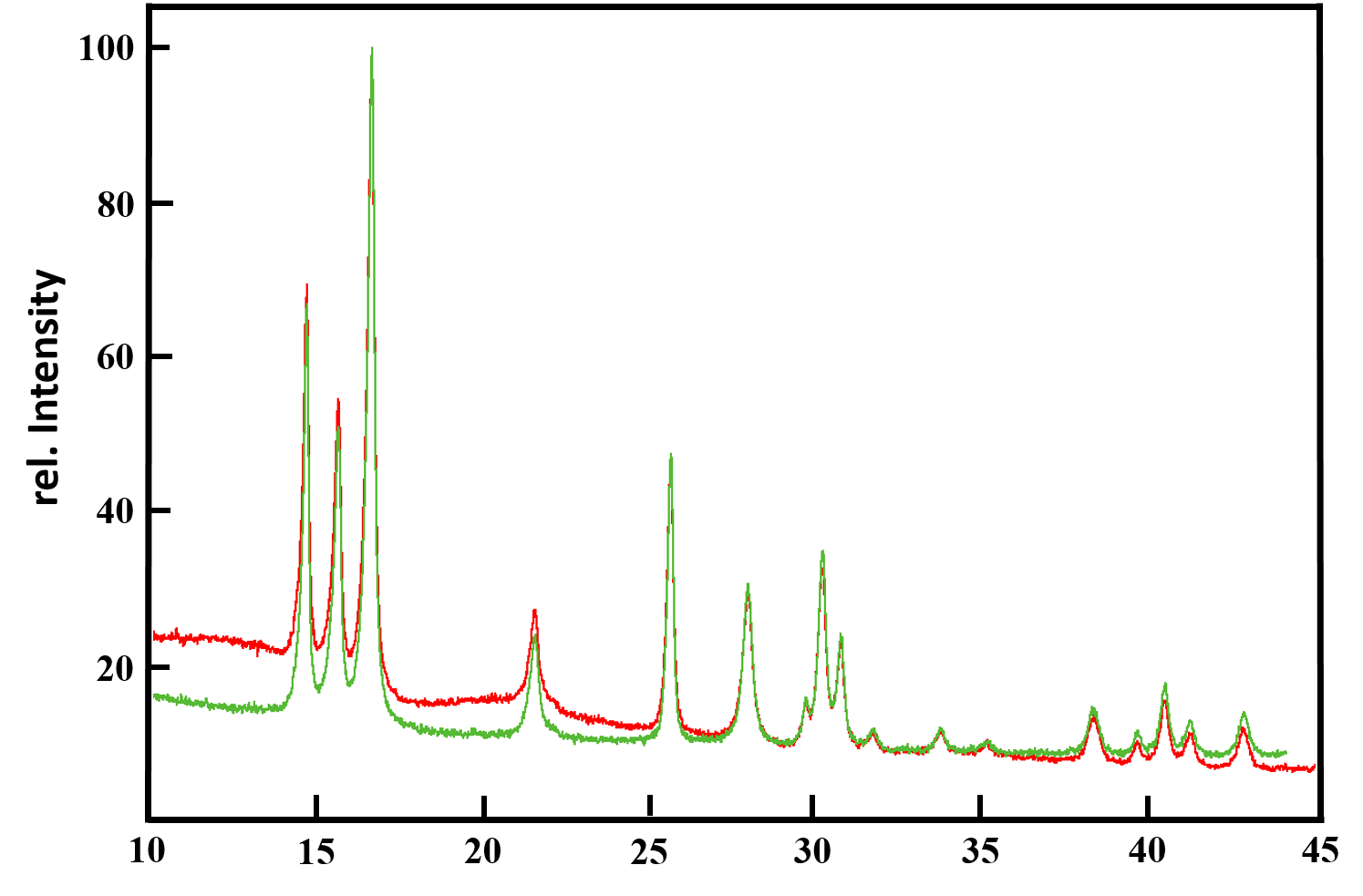


Figure S1: X-ray powder diffraction pattern of GaN:Eu (red) and GaN:Tb (green), (*λ* = 0.709026 Å).


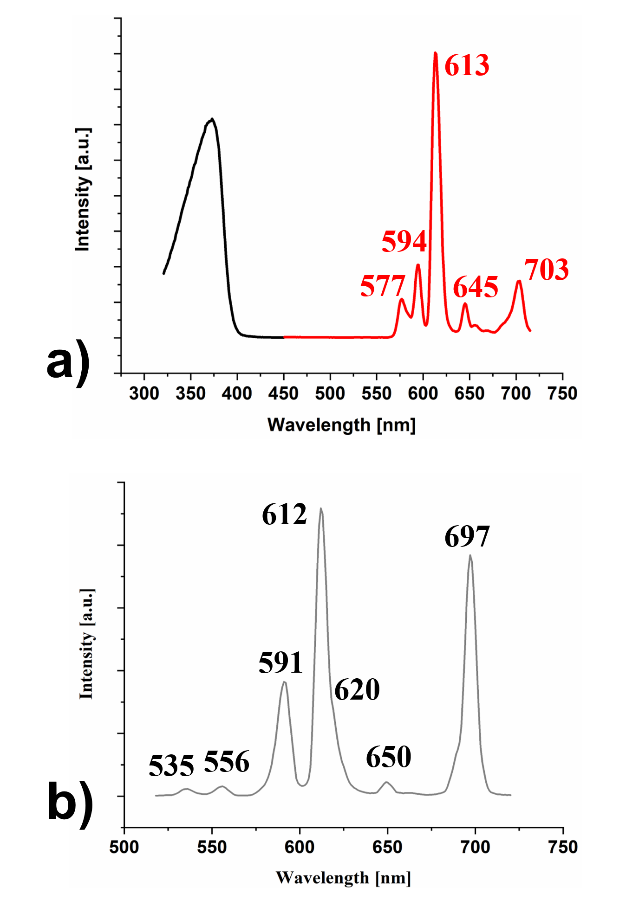


Figure S2: Luminescence spectra of a) GaN:Eu and b) EuCl_3_ x 6 H_2_O.


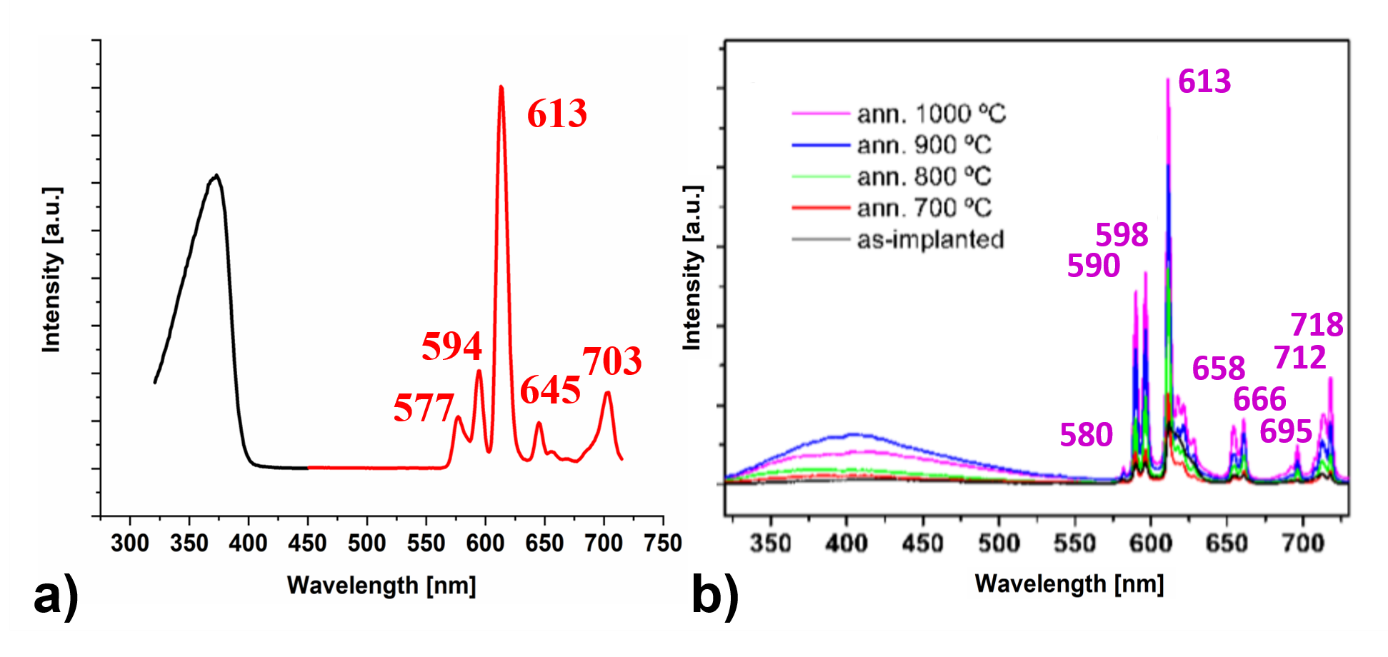


Figure S3: Luminescence spectra of a) GaN:Eu and b) β-Ga_2_O_3_:Eu.^1^ (Wavelengths are estimated from the scale in *ref.*^1^)


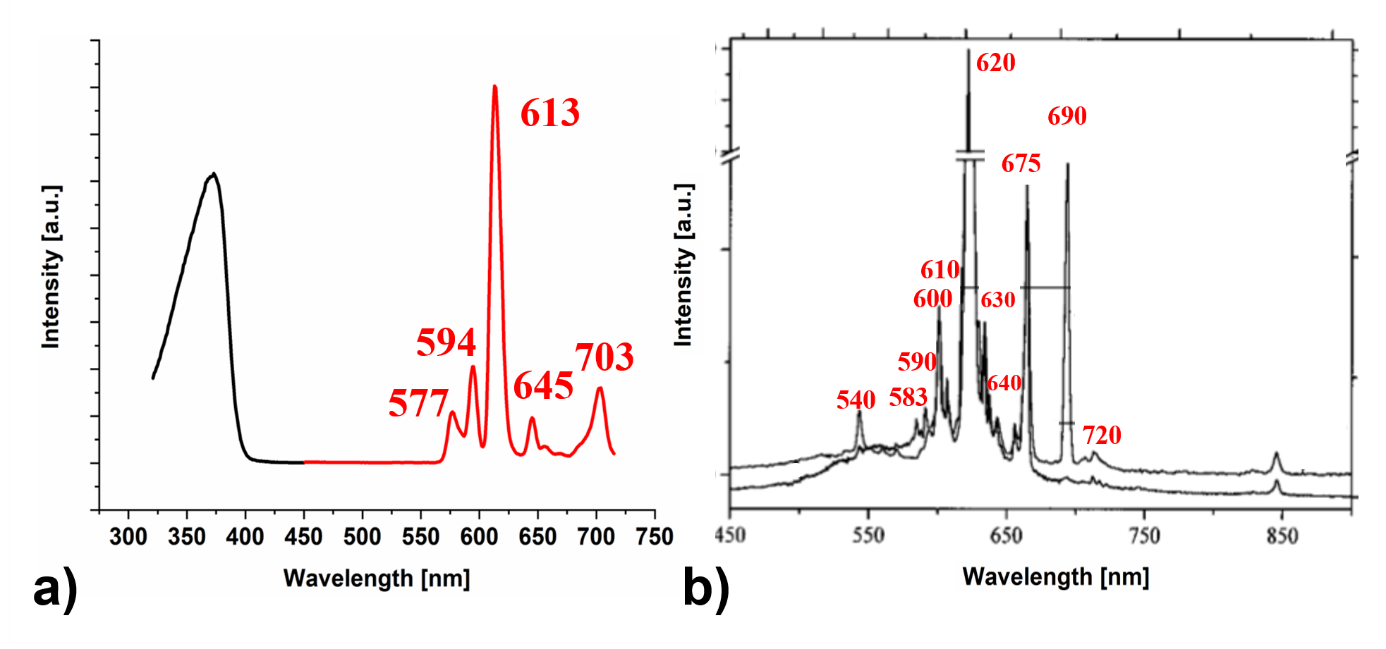


Figure S4: Luminescence spectra of a) GaN:Eu and b) GaN:Eu from a metalorganic vapor phase epitaxy growth.^2^ (Wavelengths are estimated from the scale in *ref.*^2^)

**
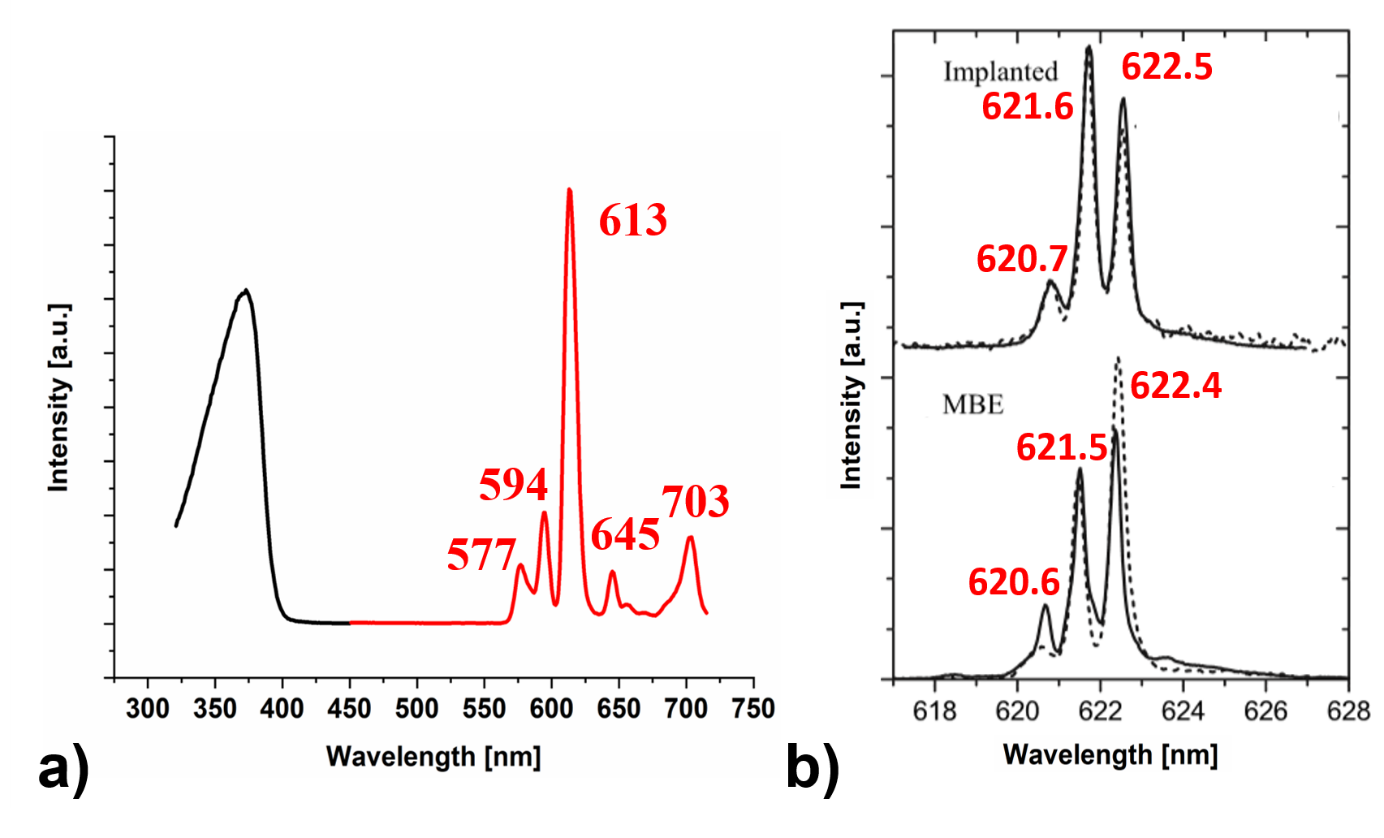
**

Figure S5: Luminescence spectra of a) GaN:Eu and b) GaN:Eu from Eu doped GaN thin ﬁlms and Eu doped GaN Quantumdots.^3^ (Implanted: sample implanted with Eu ions, MBE: Molecular beam epitaxy) (Wavelengths are estimated from the scale in *ref.*^3^)


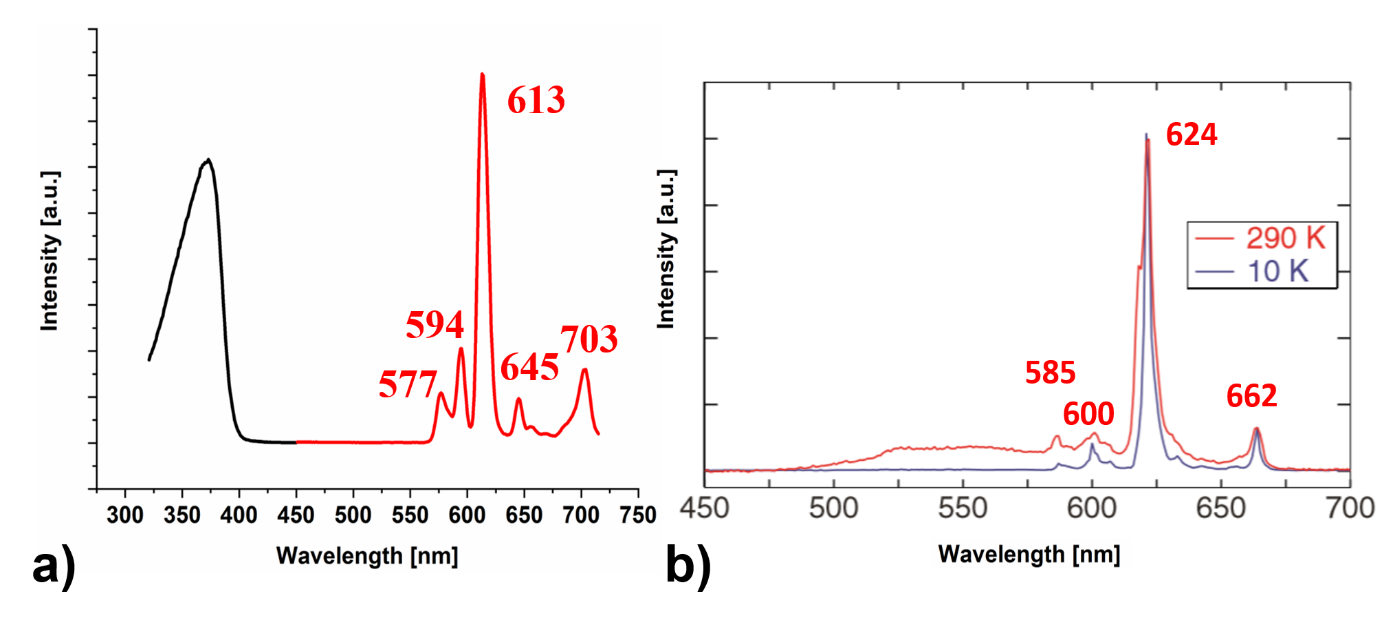


Figure S6: Luminescence spectra of a) GaN:Eu and b) GaN:Eu grown from thin layers by organometallic vapor-phase epitaxy.^4^ (Wavelengths are estimated from the scale in *ref.*^4^)


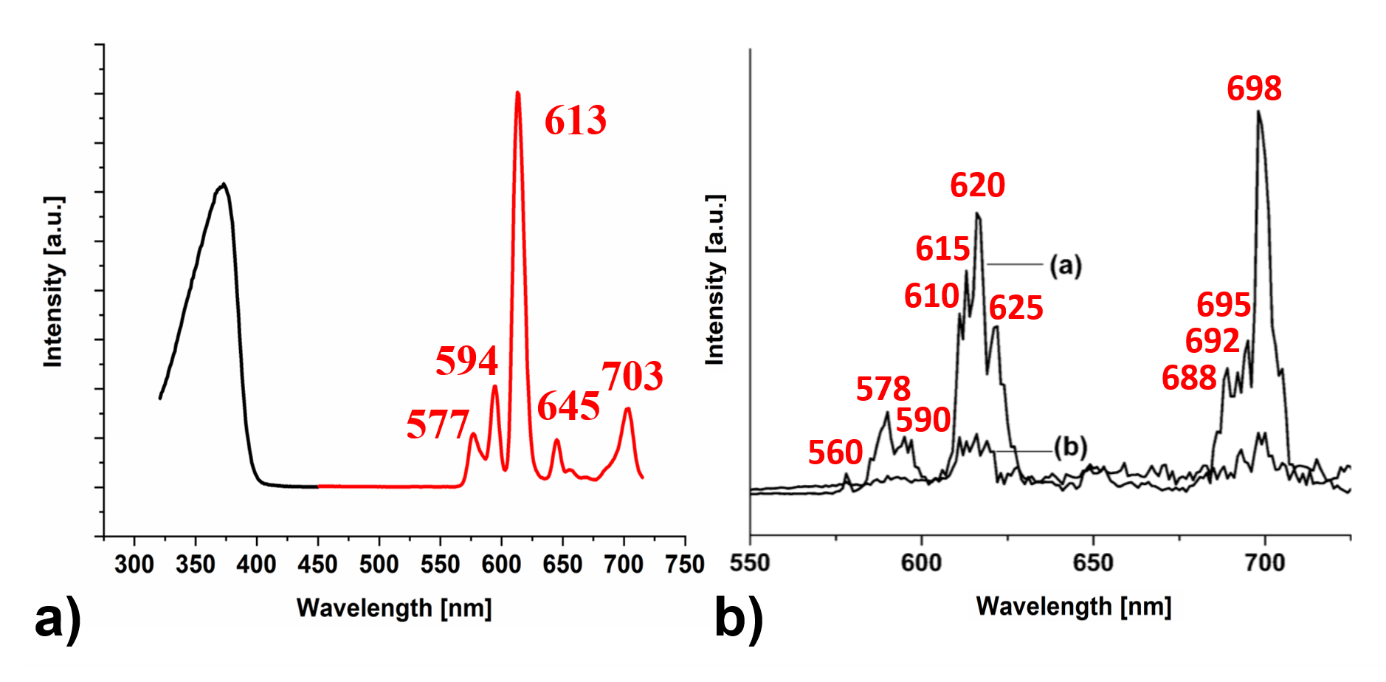


Figure S7: Luminescence spectra of a) GaN:Eu and b) GaN/SiO_2_:Eu Nanocomposites.^5^

((a) Eu^3+^-doped Ga_2_O_3_/SiO_2_, b) the same after nitridation in ammonia at 900°C^5^) (Wavelengths are estimated from the scale in *ref.*^5^)


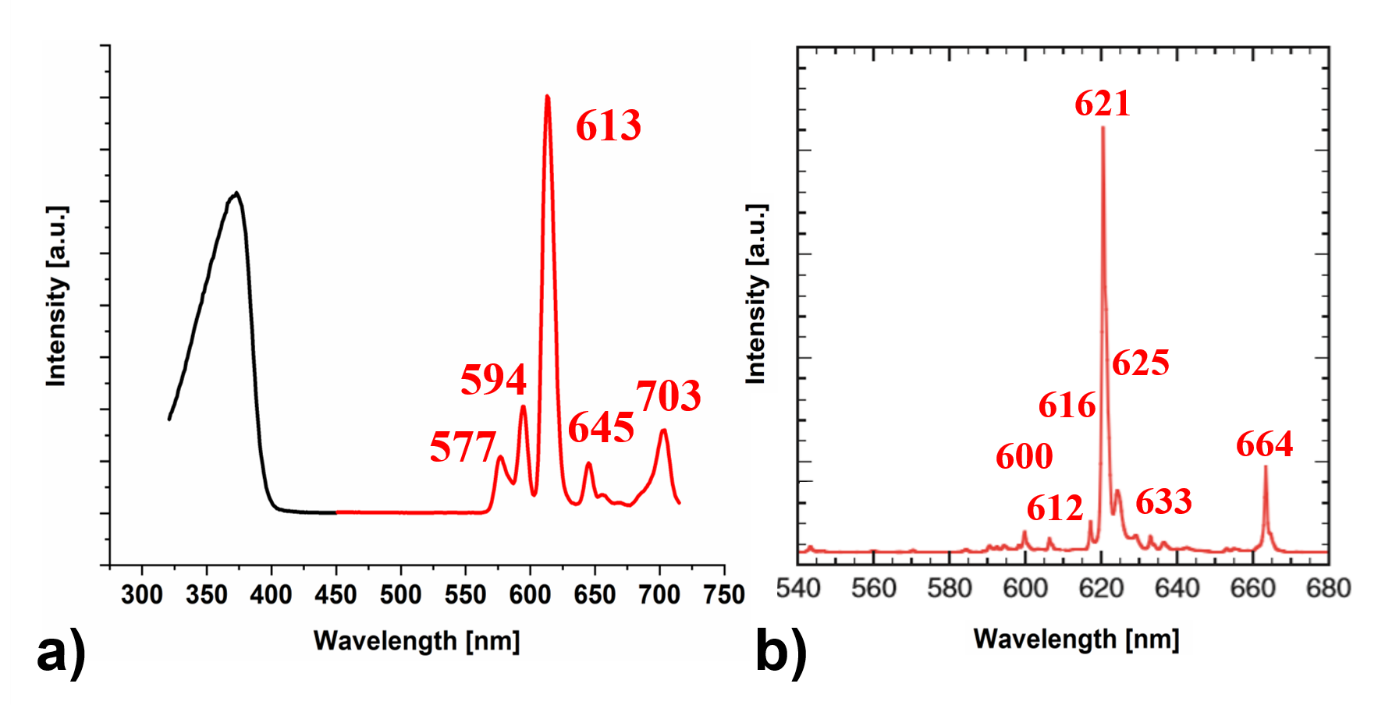


Figure S8: Luminescence spectra of a) GaN:Eu and b) GaN:Eu layer grown by organometallic vapor-phase epitaxy.^6^ (Wavelengths are estimated from the scale in *ref.*^6^)


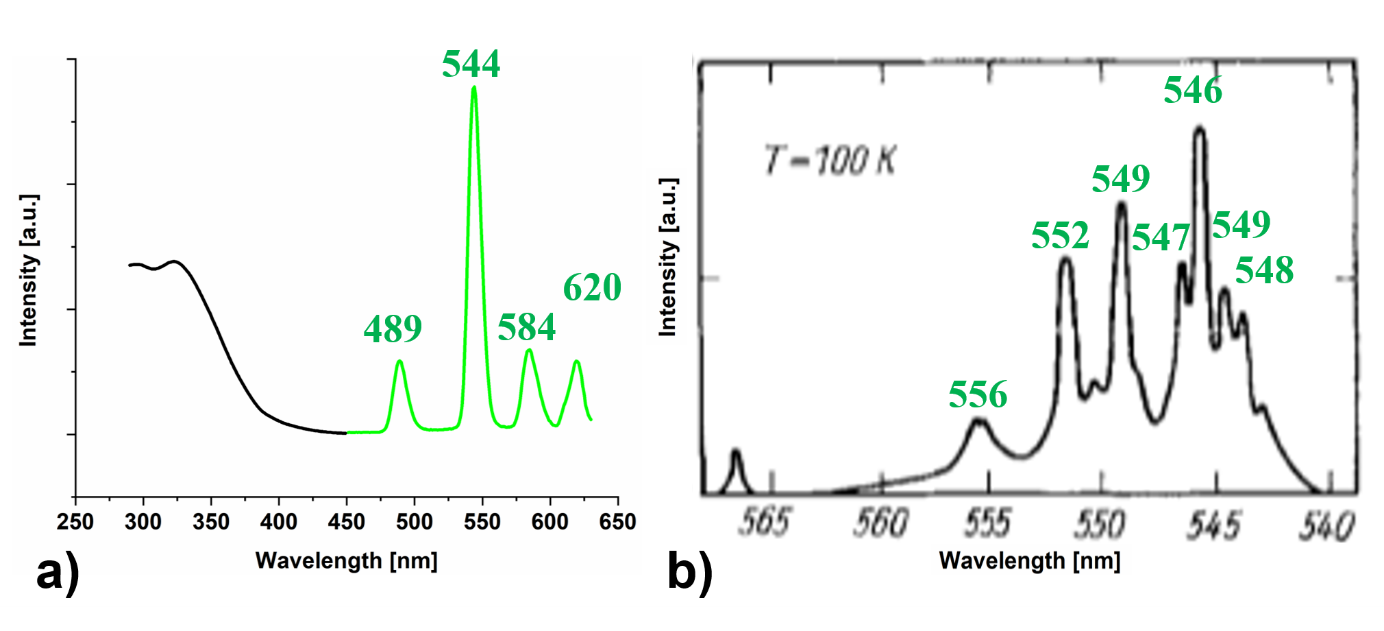


Figure S9: Luminescence spectra of a) GaN:Tb and b) TbOCl.^7^ (Wavelengths are estimated from the scale in *ref.*^7^)


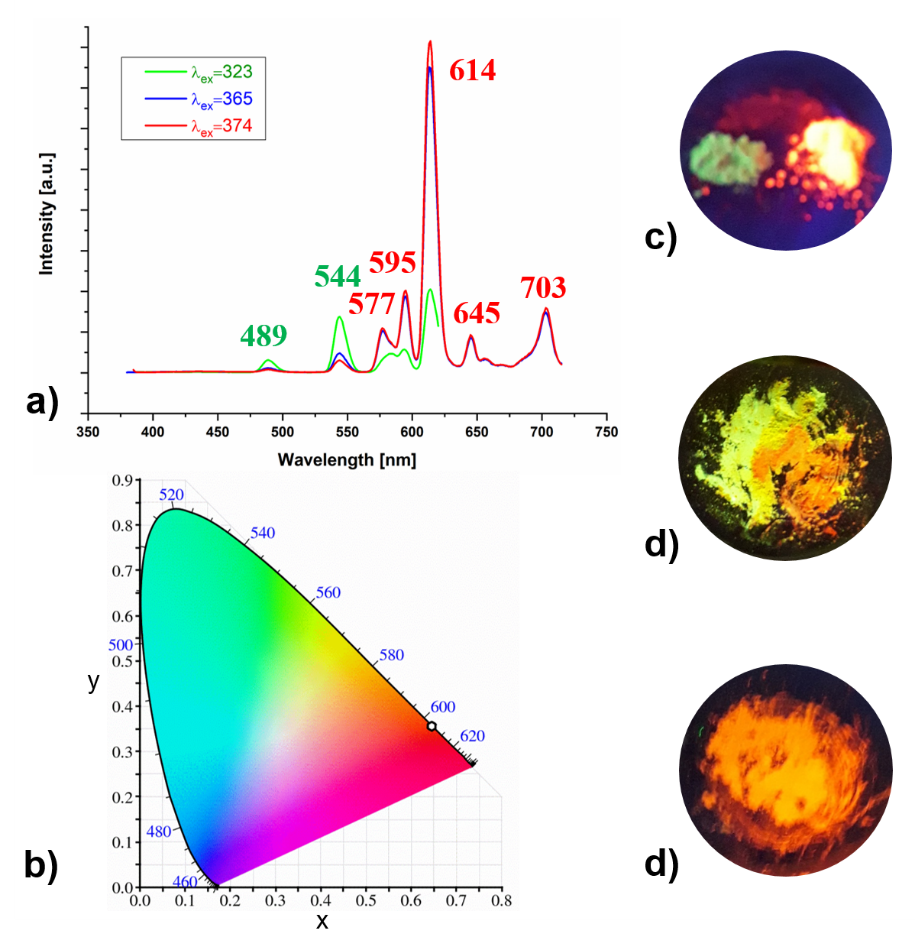


Figure S10: a) Luminescence spectra of GaN:Eu and GaN:Tb powders mixed in a mortar, at measured at different wavelengths (323, 365, 374 nm), b) CIE Diagramm of mixed GaN:Eu and GaN:Tb powders, c) GaN:Eu and GaN:Tb side by side in a mortar, d) starting the mixing procedure of GaN:Eu and GaN:Tb, e) mixed GaN:Eu and GaN:Tb.

***References***

1. Peres, M. *et al.* Doping β-Ga_2_O_3_ with europium: Influence of the implantation and annealing temperature. *J. Phys. D. Appl. Phys.* **50**, 325101 (2017).

2. Lozykowski, H. J., Jadwisienczak, W. M., Han, J. & Brown, I. G. Luminescence properties of GaN and Al_0.14_Ga_0.86_N/GaN superlattice doped with europium. *Appl. Phys. Lett.* **77**, 767–769 (2000).

3. Bodiou, L. *et al.* Optically active centers in Eu implanted, Eu *in situ* doped GaN, and Eu doped GaN quantum dots. *J. Appl. Phys.* **105**, 043104 (2009).

4. de Boer, W. D. A. M. *et al.* Optical excitation and external photoluminescence quantum efficiency of Eu^3+^ in GaN. *Sci. Rep.* **4**, 5235 (2015).

5. Mahalingam, V. *et al.* Bright Blue Photo- and Electroluminescence from Eu^2+^-Doped GaN/SiO_2_ Nanocomposites. *Adv. Funct. Mater.* **17**, 3462–3469 (2007).

6. Nishikawa, A., Kawasaki, T., Furukawa, N., Terai, Y. & Fujiwara, Y. Room-Temperature Red Emission from a p-Type/Europium-Doped/n-Type Gallium Nitride Light-Emitting Diode under Current Injection. *Appl. Phys. Express* **2**, 071004 (2009).

7. Berdowski, P. A. M., van Herk, J., Jansen, L. & Blasse, G. Luminescence and Energy Transfer Characteristics of TbOCl. *Phys. status solidi* **125**, 387–391 (1984).
